# Physiological and biochemical response of strawberry cv. diamond to nano zeolite soil application and cinnamic acid foliar application

**DOI:** 10.1038/s41598-024-76419-5

**Published:** 2024-11-21

**Authors:** Najmeh Zeinalipour, Safoora Saadati

**Affiliations:** Department of Horticultural Science, Faculty of Agriculture, Shahid Bahonar University of Kerman, P.O. Box: 76169-133, Kerman, Iran

**Keywords:** Antioxidants, Cinnamic acid, Fruit quality, Strawberry, Nano fertilizer, Yield, Plant physiology, Fertilization

## Abstract

The increasing global population and climate change are pressing challenges that necessitate innovative agricultural solutions to ensure food security. One promising approach is the use of nano fertilizers and foliar spraying techniques, which enhance nutrient delivery to plants. The purpose of this research was to examine how three different concentrations of nano zeolite (0, 30, and 60 mg L^− 1^) applied to the soil through irrigation and three different concentrations of cinnamic acid (0, 100, and 200 µM) applied topically affected the physiological and biochemical characteristics of strawberry (*Fragaria* × *ananassa* Duch.) cv. diamond, as well as the crop’s yield and quality of its fruits. A factorial system with three replications was used in the experiment, which was designed in a completely randomized design. According to the data, all features except stomatal conductivity were considerably impacted by soil application of nano zeolite and foliar spraying of cinnamic acid. Moreover, cinnamic acid (200 µM) spray combined with soil application nano zeolite (60 mg L^− 1^) decreased electrolyte leakage, transpiration rate, and malondialdehyde, while enhanced chlorophyll content, net photosynthesis rate, leaf dry matter, relative water content, fruit length and diameter, yield, and fruit quality parameters.

**Supplementary Information:**

The online version contains supplementary material available at 10.1038/s41598-024-76419-5.

## Introduction

The horticulture product known as cultivated strawberries (*Fragaria x ananassa* Duch.) is grown all over the world and is highly valuable because of its robust aroma, delicious flavor, appealing look, and copious nutritious content^1^. According to FAO statistics, the cultivated area and production rate of strawberries in Iran were 4438 h and 63605.75 t respectively^2^. Kerman province, located in southern Iran (Lat: 30° 17′ 2.1732″ N; Lang: 57° 5′ 0.1068″ E), is a major center for strawberry production and supply in the country. However, strawberry cultivation in this region faces several challenges, particularly related to soil characteristics.

New studies show that nanotechnology has the potential to completely alter farming practices^3^. An elegant, safe, target-bound, and easily delivered agrochemical distribution mechanism is made possible by this platform. Nano fertilizers outperform the majority of modern polymeric-type conventional fertilizers because of their extremely high surface area-to-volume ratio. Their characteristics may also facilitate gradual release and enhance crop nutrient uptake efficiency. According to Jakhar et al.^4^, this technology paves the way for innovative and environmentally friendly nutrient delivery systems that use the plant components’ nanoporous surfaces. Soil fertility, plant health, environmental pollution, and agroecology degradation can all be improved with the help of encapsulated nanoparticles, nanoclays, and zeolites, according to research by Manjunatha et al.^5^ Zeolites are hydrated aluminosilicates of alkaline and alkaline earth cations with a crystalline, nanoporous structure and an infinitely open three-dimensional arrangement These cations serve as inorganic soil conditioners^6^. According to Wen et al.^7^, clinoptilolite is the most abundant zeolite found in nature. Having a nanoporous structure, zeolites can hold water and nutrients, and this unique structure allows them to retain nutrients and slowly release them^8^. Applying inorganic fertilizers combined with zeolites significantly enhanced N, P, and K uptake and their usage efficiency in roots, leaves, and stems, and zeolite also altered nutrient concentrations determined in maize (*Zea mays* L.) tissues^9^. Soil conditioners containing zeolites are popular due to their beneficial impacts on soil physio-chemical parameters, which include enhancing soil moisture, hydraulic conductivity, and yields in acidified soils^10^ The use of Brazilian zeolitic sedimentary rocks as soil conditioners has shown significant benefits for agricultural productivity, particularly for crops such as lettuce, tomato, rice, and Andropogon grass^11^. Likewise, Mazur et al.^12^ found that 15 tones ha^− 1^ of clinoptilolite increased potato, barley, and clover yields, while Bouzo et al.^13^ found that sugar cane yields increased with applying 6 tones ha^− 1^. According to Gholamhoseini et al.^14^, zeolites increase water-use efficiency (WUE) by making more water available to crops and enhancing the soil’s capacity to hold water. Gholizadeh-Sarabi and Sepaskhah^15^ found that improving soil-hydraulic characteristics boosted agricultural output and water use efficiency. Zeolites can effectively adsorb carbon dioxide (CO_2_) from various gas mixtures, such as flue gas and biogas. Once zeolites have absorbed CO_2_, they can release it slowly back into the environment under certain conditions^16^. The concentration of carbon dioxide close to the plant’s stomata may be increased by spraying zeolites onto the leaves. Because of this phenomenon, C_3_ plants (e.g., vines, tomato plants, apple trees, and orange trees) may boost their photosynthetic rate by reducing carbon dioxide loss through photorespiration and increasing the efficiency of net carbon CO_2_ intake. This leads to higher growth, an increase in the leaf surface production rate, and a decrease in transpiration rate^17^.

Most secondary metabolites linked to plant allelopathy in natural and agroecosystems are phenolic compounds, a class of natural chemicals mostly produced by plants^18^. Allelopathic phenomena are mediated by the most biologically active molecules in this class, which include cinnamic acid, chlorogenic acid, and other caffeic acid esters^19^. Cinnamic acid has been shown to have significant beneficial effects on plant growth and productivity. According to a study by Talaat and Balbaa^20^, the phenylpropanoid cis-cinnamic acid promotes plant development in general, whereas the trans-isomer has no effect at equimolar doses. Cinnamic acid’s effects, however, can differ according to its concentration and type. According to Khawula et al.^21^, the mechanism of action used by hydroxycinnamic acid involves the minimization of oxidative damage to maintain cellular homeostasis and protect vital cellular components from harm. Although cinnamic acid has been shown to promote plant development, it’s worth noting that it can impede other growth processes, like disease resistance and flavonoid exudation^22^.

To our knowledge, there is no direct evidence of studies on the exogenous application of cinnamic acid and nano zeolite on strawberries. This study investigated the effect of different concentrations of foliar application of cinnamic acid and nano zeolite used in soil on growth characteristics, yield, and quality of strawberries (*Fragaria × ananassa* Duch.) cv. Diamond.

## Results

### Electrolyte leakage and malondialdehyde

 The results obtained from Table 1 showed that the simple and interaction effects of cinnamic acid and nano zeolite were significant physiological and biochemical parameters of strawberry leaves cv. diamond. The highest electrolyte leakage was obtained in the control treatment (49.67%), while its lowest was observed in the interaction effect of cinnamic acid 200 µM without nano zeolite (11.08%), although no significant difference was observed between this treatment with cinnamic acid 200 µM + nano zeolite 60 mg L^− 1^ (13.5%) (Table 2).

The results shown in Table 2 indicate that the highest amount of malondialdehyde (µmol g^− 1^ FW) was observed in the control treatment (0.37 µmol g^− 1^ FW), while the lowest was found in the treatment of 100 and 200 µM cinnamic acid without nano zeolite (0.13 and 0.14 µmol g^− 1^ FW, respectively).

### Chlorophyll content

 Findings of Table 2 showed that the lowest amount of chlorophyll a was related to the control treatment (11.16 mg g^− 1^ FW), however the highest was related to the combined treatment of cinnamic acid 200 µM and nano zeolite 60 mg L^− 1^ (14.26 mg g^− 1^ FW), which was almost 26% more than the control. The lowest and highest amount of chlorophyll b was related to the control treatment and the combined treatment of cinnamic acid 200 µM and nano zeolite 60 mg L^− 1^ (8.19 and 11.81 mg g^− 1^ FW, respectively). The lowest amount of total chlorophyll was observed in the control treatment (19.35 mg g^− 1^ FW), and the highest amount of total chlorophyll was found in the treatment of the interaction of cinnamic acid 200 µM + nano zeolite 60 mg L^− 1^ (26.07 mg g^− 1^ FW), which was almost 34% more than the control.

### Stomatal conductance, transpiration rate, and net photosynthetic rate

 The results of the analysis of variance and means comparison showed that there was no significant difference between the treatments on the amount of leaf stomatal conductance (Tables 1 and 2).

**Table 1:** Variance analysis of the effect of cinnamic acid (CA) and nano zeolite (NZ) on the physiological and biochemical parameters of the leaves of the strawberry cv. Diamond. ^Ns, *, **, ***^nonsignificant or significant at *P* ≤ 0.05, 0.01, 0.001 , respectively.

Source of variation	df	Mean of squares									
		Electrolyte leakage	Malondialdehyde	Chlorophyll a	Chlorophyll b	Total chlorophyll	Stomatal conductance	Transpiration rate	Net photosynthesis rate	Leaf dry matter	Relative water content
CA	2	0.095^*^	0.054^*^	12.677 ^***^	10.90 ^***^	47.257 ^***^	0.003 ^ns^	0.282 ^***^	1149.01^**^	256.3 ^*^	419.17 ^**^
NZ	2	0.123^**^	0.098^*^	5.471 ^***^	21.64 ^***^	48.500 ^***^	0.001 ^*^	0.279 ^***^	162.23 ^*^	82.45 ^ns^	611.24 ^**^
CA × NZ	4	0.0142^*^	0.051 ^*^	0.844 ^*^	3.284 ^***^	4.692 ^***^	0.000 ^ns^	0.206 ^***^	0.097 ^*^	6.77 ^*^	51.55 ^*^
Error	18	0.0041	0.0021	0.266	0.329	0.510	0.000	0.043	293.01	5.56	12.62
CV (%)	-	6.46	5.47	4.21	5.7	3.21	14.8	18.01	10.58	7.23	7.861

**Table 2:** The physiological and biochemical parameters of strawberry leaves affected by cinnamic acid and nano zeolite. Means followed by the similar letters in each common are not statistically different (*P* ≤ 0.05) as compared to the LSD test.

Treatments		Electrolyte Leakage (%)	Malondialdehyde (µmol g^− 1^ FW)	Chlorophyll a (mg g^− 1^ FW)	Chlorophyll b (mg g^− 1^ FW)	Total chlorophyll (mg g^− 1^ FW)	Stomatal conductance (mmol m^− 2^ s^− 1^)	Transpiration rate (µmol H_2_O_2_ m^− 2^ s^− 1^)	Net photosynthesis rate (µmol CO_2_ m^− 2^. s^− 1^)	Leaf dry matter (g m^− 2^)	Relative water content (%)
Cinnamic acid (µM)	Nano zeolite (mg L^− 1^)										
0	0	49.67 a	0.37 a	11.16 e	8.19 c	19.35 c	0.04 a	1.40 a	14.63 d	489 e	66.89 c
	30	28.92 b	0.30 b	11.26 e	8.34 c	19.40 c	0.04 a	1.28 ab	15.34 c	511 d	67.31 c
	60	25.64 b	0.30 b	12.21 cd	11.43 a	23.64 b	0.06 a	0.09 cde	18.4 b	544 c	84 b
100	0	21.33 c	0.13 e	11.63 de	7.83 c	19.47 c	0.04 a	0.66 de	15.16 c	505 d	75.89 d
	30	26.6 b	0.15 d	10.27 f	8.18 c	18.44 c	0.06 a	0.91 cd	17.3 b	564 b	78.12 d
	60	19.62 c	0.16 d	12.91 bc	12.00 a	24.92 a	0.05 a	0.95 bcd	18.37 b	571 b	87.71 b
200	0	11.08 d	0.14 e	13.23 b	11.62 a	24.85 ab	0.06 a	1.21 abc	21.08 a	568 b	85.67 b
	30	16.67 c	0.18 c	13.42 ab	10.27 a	23.69 b	0.08 a	1.09 abc	22.48 a	645 ab	86.01 b
	60	13.5 d	0.15 d	14.26 a	11.81 a	26.07 a	0.09 a	0.53 e	23.15 a	674 a	90.01 a

The results of the comparison of means showed that the lowest and highest transpiration rates were related to cinnamic acid 200 µM + nano zeolite 60 mg L^− 1^ and control treatments (0.53 and 1.40 µmol H_2_O_2_ m^− 2^ s^− 1^, respectively) (Table 2). The results of Table 2 indicated that the lowest Net photosynthesis rate was in the control treatment (14.63 µmol CO_2_ m^− 2^. s^− 1^), while the highest rate was observed in the cinnamic acid 200 µM + nano zeolite 60 mg L^− 1^ treatment (23.15 µmol CO_2_ m^− 2^. s^− 1^). No statistically significant difference was observed between cinnamic acid 200 µM treatment and all nano zeolite levels.

### Leaf dry matter

 The highest and lowest leaf dry matter was observed in the treatments of the interaction of cinnamic acid 200 µM + nano zeolite 60 mg L^− 1^ and the control treatment, respectively. However, there was no statistically significant difference in this trait between the cinnamic acid 200 µM with nano zeolite 30 and 60 mg L^− 1^ (Table 2).

### Relative water content

 The comparison of the means relative water content showed that the lowest and highest values were found in control (66.89%) and cinnamic acid 200 µM + nano zeolite 60 mg L^− 1^ (90.01%) treatments, respectively, however there was no significant difference between the control and nano zeolite 30 mg L^− 1^ without cinnamic acid treatments (Table 2).

### Length and diameter of fruit

 The results of the analysis of the variance of the data showed that the interaction of cinnamic acid and nano zeolite was significant on the quantitative and qualitative traits of strawberry fruit cv. diamond (Table 2).

The comparison of means showed that the maximum and minimum length and diameter of strawberry fruit were related to cinnamic acid 200 µM + nano zeolite 60 mg L^− 1^ and control treatments, respectively. However, no significant difference was observed between the treatments of cinnamic acid 200 µM with all levels of nano zeolite (Table 3).

**Table 3:** Variance analysis of the effect of cinnamic acid (CA) and nano zeolite (NZ) on the quantitative and qualitative parameters of the strawberry cv. Diamond. ^Ns, *, **, ***^nonsignificant or significant at *P* ≤ 0.05, 0.01, 0.001 , respectively.

Source of variation	df	Mean of squares								
		Fruit length	Fruit diameter	Fruit firmness	Yield	TSS	TA	Antioxidant activity	Anthocyanin	Ascorbic acid
CA	2	7.6^ns^	256.3^**^	11.56*	24545.2**	492.04**	0.0028**	326.62*	23.44*	412.09*
NZ	2	8.3*	10.11^ns^	328.9**	3344.21**	3213.39*	0.000022 ^ns^	463.0**	111.7^ns^	267.1^ns^
CA × NZ	4	0.05*	6.44*	0.09*	113.28*	30,719. 3**	0.00028**	0.3245*	261.9 *	276.5*
Error	18	5.17	11.96	11.2	15.734	451.25	0.000004	172.6	0.07	14.49
CV (%)	–	11.3	10.47	8.44	4.831	3.0295	5.2196	8.19	9.1	6.3

### Fruit firmness, TSS, TA, and yield

 The highest level of fruit firmness, TSS, and TA was observed in the interaction effect of cinnamic acid 200 µM with all levels of nano zeolite (0, 30, and 60 mg L^− 1^), while their lowest was obtained in the interaction effect without cinnamic acid (0 µM) with all levels of nano zeolite (Table 4).

The highest and lowest fruit yields were obtained in the treatments of the interaction of cinnamic acid 200 µM + nano zeolite 60 mg L^− 1^ (509.89 g/plant) and the control (293.12 g/plant), respectively (Table 4).

### Antioxidant activity, anthocyanin, and ascorbic acid

 The results shown in Table 4 indicated that the lowest amount of antioxidant activity, anthocyanin, and ascorbic acid were present in the control treatment, but most of them belonged to the interaction treatment of cinnamic acid 200 µM + nano zeolite 60 mg L^− 1^.

## Discussion

In this study, cinnamic acid and nano zeolite effectively decreased EL and MDA in the leaves of strawberry cv. diamond (Table 2). According to our research, it has been found that cinnamic acid decreased MDA levels in maize^23^ and sweet pepper^24^ seedlings under salinity stress, and alleviated the adverse effects of NaCl derivative. In line with current results, Li et al.^25^ claimed that cinnamic acid restored plasma membrane function by reducing lipid peroxidation in cucumber leaves. A study by Gill et al.^26^ investigated the effects of a coating made from gum Arabic combined with cinnamic acid on mango fruits during cold storage. Their results indicated that this coating significantly reduced electrolyte leakage compared to uncoated mangoes.

One study found that canola cv. Zarfam had the best biological yield and the least amount of electrolyte leakage when treated with zeolite^27^. Recent research on tomato plants irrigated with sewage water showed that the addition of zeolite significantly reduced MDA levels and lead accumulation in the plants^28^. These findings suggest that zeolite may have antioxidant effects and can help reduce oxidative stress, as evidenced by the decreased EL and MDA levels in the studies. The exact mechanisms of zeolite’s antioxidant effects are not well known, but it is suggested that zeolite’s ability to adsorb various compounds and its cation-exchange capacity may play a role in reducing MDA levels and oxidative stress^29^.

Table 2 showed an effective increase in chlorophyll and net photosynthesis rate after being treated with cinnamic acid 200 µM + nano zeolite 60 mg L^− 1^. The effects of cinnamic acid on chlorophyll and photosynthesis depend on various factors such as concentration, plant species, and experimental conditions. Cinnamic acid has been shown to significantly impact the photosynthetic efficiency and quantum yield in *Lactuca sativa* L. In a study examining the effects of various concentrations of cinnamic acid, it was found that treatment with 1.5 mM cinnamic acid drastically reduced several key parameters associated with photosynthesis^30^. Another study found that 25 μm L^− 1^ of cinnamic acid had a promotion effect on chlorophyll a and b in *Cucumis sativus* seedlings, while 150 μm L^− 1^ of cinnamic acid had a significant inhibition effect on chlorophyll a and b^31^. According to Yang et al.^32^, intercropping faba beans and wheat significantly mitigate the effects of cinnamic acid toxicity on faba bean photosynthetic physiology, ultimately reducing the incidence of Fusarium wilt. Cinnamic acid-induced stress significantly reduced the amounts of chlorophyll a, chlorophyll b, chlorophyll a + b, and carotenoids in cucumber seedlings, according to another study^33^.

Based on our findings, it has been suggested that zeolite treatment can improve photosynthetic parameters, chlorophyll concentration, and photosystem II efficiency, which in turn enhances photosynthesis^34^. Zeolites are believed to influence photosynthesis by adsorbing CO_2_, reducing leaf temperature, and improving water-use efficiency, which can positively impact plant growth and productivity^35^.

In our research, the combined treatment of cinnamic acid and nano zeolite significantly increased the RWC of strawberry cv. diamond (Table 2). In line with our results, has shown that cinnamic acid can increase the RWC and dry matter in plants, indicating its potential to improve water retention and biomass production^23^. The use of exogenous silicon reversed the effect of cinnamic acid-induced autotoxicity stress on cucumber seedlings, leading to an increase in RWC, contrary to the original finding^33^. Additionally, in banana leaves, cinnamic acid enhanced antioxidant activities, soluble sugar levels, and RWC under heat stress, and decreased lipid peroxidation, indicating its role in mitigating oxidative stress and contributing to the recovery of growth and net CO_2_ assimilation after heat stress^36^. According to recent research, cinnamic acid enhanced photosynthesis by increasing the antioxidant system activity and proline accumulation, while decreasing Na^+^ and Cl^−^ levels and lipid peroxidation^24^.

In our research, the cinnamic acid and nano zeolite treatments significantly enhanced leaf dry matter (Table 2). Similar to our findings, it has been observed that zeolite increases the accumulation of dry matter in all kinds of plants and plant species^37^. Research has consistently shown that applying zeolite positively impacts plant development and growth, particularly by increasing dry matter accumulation across various growth stages. This impact has been seen in rice, lettuce, and tomatoes^38^. Application of zeolite on *Aloe vera* L. plants improved their development and yield while reducing the negative impacts of water stress^39^.

In this research, quantitative fruit traits such as length, diameter, and yield were significantly affected by cinnamic acid and nano zeolite treatments (Table 4). In parallel with our results, research has shown that cinnamic acid and its derivatives have been found to enhance tomato resilience, stimulate growth, and increase fruit yield^40^. Cinnamic acid autotoxicity lowered plant growth and yield, impeded element absorption, and drastically decreased relative water content, which contradicts our findings ^33,41^.

In line with our results, research has demonstrated that zeolite application can increase grain yield and mitigate greenhouse gas emissions in rice fields^42^. In addition to improving growth, yield characteristics, and yield, zeolite has been found to promote soil health, agricultural production, and environmental safety^43^. Furthermore, the application of zeolite in combination with other organic inputs has been found to have a favorable impact on the quantitative and qualitative features of Black cumin (*Nigella sativa* L.), including increased seed yield and oil production^44^. Table 4 shows that cinnamic acid, particularly at 200 µM in the compound with all levels of nano zeolite, enhanced the strawberry taste by influencing the TSS and TA of the fruit. Consistent with our findings, applying of cinnamic acid has been reported to improve the TSS and TA in various fruits. A study on tomatoes treated with chitosan and cinnamic acid showed an improvement in quality attributes such as firmness and total soluble solids while reducing physiological aspects^45^. Another study mentioned the role of cinnamic acid in improving berry quality and its impact on TSS and TA^46^. Furthermore, research on jujubes demonstrated that an edible coating enriched with cinnamic acid led to an increase in total soluble solids, indicating a potential impact on TSS^47^. Moreover, researchers have demonstrated that zeolite can significantly improve the firmness of table grapes, enhance different phytochemicals, and increase TSS while minimizing fruit acidity^48^. According to Harhash et al.^49^, zeolite can increase nutrient conservation, decrease soil acidity, and improve the quality and production of mango fruits. The incorporation of zeolite into soil can lead to better nutrient retention and availability, which is crucial for optimal fruit development and quality^50^.

**Table 4:** The quantitative and qualitative parameters of strawberry fruit affected by cinnamic acid and nano zeolite. Means followed by the similar letters in each common are not statistically different (*P* ≤ 0.05) as compared to the LSD test.

Treatments		Fruit length (mm)	Fruit diameter (mm)	Fruit firmness (N cm^− 2^)	Yield (g/plant)	TSS (%)	TA (%)	Antioxidant activity (%)	Anthocyanin (µg g^− 1^ FW)	Ascorbic acid (mg/100 g FW)
Cinnamic acid (µM)	Nano zeolite (mg L^− 1^)									
0	0	48.86 c	37.78 e	11.37 d	293.12 f	7.79 d	3.82 d	3.70 b	14.36 f	27.50 e
	30	51.13 bc	39.45 de	15.8 d	345.34 e	7.90 d	3.90 d	3.8 b	14.48 f	28.20 e
	60	53.20 b	39.72 de	15.45 d	389.15 d	7.74 d	3.39 d	3.80 b	16.22 e	29.00 d
100	0	50.43 bc	41.75 d	18.28 b	328.65 e	8.40 c	4.47 c	3.60 b	21.23 d	31.10 cd
	30	54.27 b	43.20 cd	18.27 b	423.50 cd	9.09 b	5.38 b	4.00 a	23.75 c	32.70 c
	60	60.10 ab	46.10 bc	17.97 c	437.35 c	9.18 b	4.18 c	4.10 a	23.98 c	33.20 c
200	0	60.27 ab	49.40 ab	21.65 a	345.87 e	9.27 a	6.53 a	3.70 b	19.62 d	35.10 b
	30	62.50 a	48.60 ab	21.98 a	448.90 b	9.25 a	6.55 a	4.30 a	27.35 b	37.10 a
	60	63.41 a	50.65 a	21.8 a	509.89 a	9.40 a	6. 90 a	4.50 a	30.09 a	37.80 a

Based on our findings, cinnamic acid 200 µM in the compound with nano zeolite 60 mg L^− 1^ treatment effectively enhanced antioxidant activity in strawberry fruits cv. diamond (Table 4).

There have been reports of antioxidant properties in cinnamic acid and its derivatives. Cinnamic acid and its derivatives are recognized for their significant antioxidant properties, particularly those with hydroxyl groups in the cinnamoyl moiety. These compounds are known to exhibit strong free radical scavenging abilities, which contribute to their health benefits^51,52^. Moreover, according to López-González et al.^53^, cinnamic acid and its derivatives can boost the antioxidant activity of *Zea mays* L. roots.

Research indicates that zeolite can positively influence plant antioxidant activity, particularly under drought-stress conditions. Studies have demonstrated that the application of zeolite enhances the activity of antioxidant enzymes in plants, thereby mitigating the adverse effects of drought^54^, which confirms our results. A pot experiment demonstrated that both nano zeolite and ordinary zeolite effectively decreased cadmium concentrations in the shoots and roots of two varieties of Chinese cabbage^55^. A study by Saeed et al.^56^ investigated the effects of combining the bacterium *Enterobacter sp. MN17* with zeolite to mitigate the negative impacts of cadmium on the growth and physiological functions of *Brassica napus* (rapeseed). Notably, the antioxidant enzyme activities in the plants were decreased by 25–64% when treated with this combination, which contradicts our findings.

In our research, the anthocyanin content of fruits was significantly affected by cinnamic acid and nano zeolite treatments (Table 4). Cinnamic acid has been found to have varying effects on anthocyanin stability by its concentration and the specific conditions of the system. In line with our results, a study mentioned that cinnamic acid increased color at low concentrations while inhibiting anthocyanin formation at higher concentrations^57^. Additionally, cinnamic acids have been reported to acylate anthocyanin and flavonoid glycosides, and they have higher levels of reactive oxygen scavenging activity^58^. Conversely, a study on cranberry juice found that cinnamic acid had no significant effect on anthocyanin stability during storage at ambient temperature^59^. In parallel with our findings, researchers have shown that applying zeolite, such as Zeowine, can increase anthocyanin content in grapes^60^. Zeolite-treated grapes showed increased sugar, total and extractable anthocyanin, and berry weight of *Vitis vinifera* L^60,61^.

According to our results, Araniti et al.^62^ demonstrated that applying trans-cinnamic acid to maize leaves induces stress, resulting in a metabolic shift that enhances the production of protective metabolites, including ascorbic acid, also known as vitamin C. This compound is recognized for its role in increasing plant defense mechanisms against oxidative stress and its involvement in critical processes such as cell division and phytohormone synthesis. Therefore, the increase in ascorbic acid content in plants may be a response to stress caused by trans-cinnamic acid and a part of the plant’s defense mechanism against oxidative stress^63^.

Table 4 showed an effective increase in ascorbic acid content after being treated with cinnamic acid 200 µM + nano zeolite 60 mg L^− 1^. Research indicates that applying zeolite can significantly enhance the ascorbic acid content in plants, primarily by improving the availability of essential nutrients like potassium (K) and phosphorus (P) in the growth substrate. In a study conducted by Roberts et al.^64^ it was shown that fresh tomato fruits can have a higher vitamin C content when the enriched zeolite is used as a slow-release nutrient source. This could be because the substrate has a better availability of K and P. Zeolite can indirectly influence ascorbic acid levels in coriander plants by improving their drought stress tolerance, which in turn enhances the overall growth performance and yield of the plants^65^. There may be a connection between the use of zeolite, nutrient availability, and vitamin C content in plants, however, the exact processes by which zeolite affects ascorbic acid levels remain unclear.

## Conclusion

The current research indicated that the combined foliar application of cinnamic acid at 200 µM concentration and soil application of nano zeolite at 60 mg L^− 1^ rate is the most effective way to improve physiological and biochemical parameters in strawberries. This combination reduced ion leakage and malondialdehyde levels, increased net photosynthetic rate, concentration of photosynthetic pigments, dry matter content, and improved quantitative and qualitative characteristics of the fruit. Therefore, it can be recommended to use a combination of cinnamic acid and nano zeolite to increase the yield and quality of strawberries on a larger scale, as it appears to be an effective way to improve physiological processes and fruit characteristics in strawberry plants.

## Materials and methods

### **Experimental site**

 This experiment was conducted in a plastic house with natural photoperiod conditions in Kerman province, southeastern Iran, during the 2022/2023 growth seasons. The air temperature was maintained at 25 °C and the average relative humidity was 65%. The soil of the experimental field had a sandy loam texture with a pH of 6.2. Table 5 displays the results of the chemical and mechanical investigations of the soil.

**Table 5:** Some soil physical and chemical properties of the experimental site in the growth season.

Soil property	Clay (%)	Silt (%)	Sand (%)	Textural class	pH (1:1 soil suspension)	EC (dS cm^− 1^) at 25° C	Available *N* (mg kg^− 1^ soil)	Available *P* (mg kg^− 1^ soil)	Available K (mg kg^− 1^ soil)
value	20	6	74	Sandy loam	6.2	1.32	110.31	7.70	168.00

### Preparation of plant material and treatments

 Transplants of strawberries cv. diamond was obtained from Marivan, Kurdistan, Iran. Nano zeolite (ZSM-5) with these features: unit cell size: 50 nm, SiO_2_/Al_2_O_3_ mole ratio: 15-1000, surface area: 340 m^2^ g^− 1^, Na_2_O weight: 0.05%, and nominal cation form: ammonium/hydrogen, was prepared from Pishgaman Iranian Nanomaterials Company. The cinnamomic acid was purchased from Sigma Aldrich company.

The transplants were immersed in a fosetyle-aluminum fungicide solution of 2 g L^− 1^ for 2 h, followed by thorough washing with running water to disinfect them. On November 5, 2022, planting was done on ridges of the furrow adopting the recommended spacing of 70 cm between furrows, and 25 cm between rows on the ridge. According to the conventional crop production procedures, the fertilizer solution had the following elements in milligrams per liter: N 271, P 702, K 586, Mg 207, S 414, Fe 8, Mn 4, Cu 0.3, Zn 0.8, B 0.7, and Mo 0.3 ^66^. The experiment was arranged using a factorial experiment based on a completely randomized design. The treatments include nano zeolite (ZSM-5) (0, 30, and 60 mg L^− 1^) which was supplied through the drip irrigation system, and cinnamic acid (0, 100, and 200 µM) which was applied as foliar spraying. The treatments were applied two times (at the 6–8 leaf stage and the beginning of flowering). A drip irrigation network including lateral GR with a diameter of 16 mm was allocated with a dripper at a distance of 25 cm on both sides of each row.

### Determination of leaf parameters

#### **Electrolyte leakage**

 Each test tube was supplemented with 10 mL of deionized water after the leaf discs were delicately rinsed with the water, wiped with paper, and placed in the tubes. For 24 h, the leaf sample solution was shaken at room temperature. Afterward, an electrical conductivity meter (CC-501, Elmetron, Zabrze, Poland) was used to measure the solution’s electric conductivity (EC_1_). The same samples were subsequently immersed in a water bath set to boil at 100 °C for 20 min. After the solution cooled to room temperature, the second reading (EC_2_) was calculated^67^. The electrolyte leakage was expressed as a percentage and was computed as EC_1_/EC_2_.

#### Lipid peroxidation (MDA content)

 As a measure of membrane damage, malondialdehyde (MDA) was measured as the result of lipid peroxidation in the membrane^68^. First, 0.5 g of leaves homogenized with 0.1% TCA solution and then centrifuged at 10,000 g for 10 min. Then, 4 milliliters of 20% (w/v) TCA with 0.5% (w/v) TBA was mixed with 1 milliliter of the supernatant. After 60 min of heating at 95 °C, the mixture was rapidly cooled on ice and centrifuged at 10,000 g for 10 min. Using the TCA solution as a blank for the reagent, the absorbance of the supernatant was measured at 532 and 600 nm. The MDA content was calculated with the extinction coefficient of 155 mM^− 1^ cm^− 1^.

#### Chlorophyll content determination

 One gram of fresh leaves was centrifuged in 80% acetone for 10 min to homogenize them. the supernatant was collected to measure the acetone extract absorbances at 663, 646, and 470 nm with a UV-vis spectrophotometer. the total chlorophyll content, chlorophyll a concentration, and chlorophyll b concentration were measured based on the Lichtenthaler^69^ method.

#### Stomatal conductance, transpiration rate, and net photosynthetic rate

 The measurements were made on fully grown leaves of each plant between 9:00 and 11:00 AM. An LCi 600 portable photosynthetic equipment from ADC BioScientific Ltd. in England was used to record the stomatal conductance, transpiration rate, and net photosynthetic rate.

#### Leaf dry matter

 Leaves were collected to determine leaf dry matter. The samples were weighed using an electronic scale with an accuracy of ± 0.01 g. First, the initial weight of the leaves was recorded. After that, it was placed in an oven at a temperature of 105 °C for 48 h, and their secondary weight was recorded^70^.

#### Relative water content measurement

 The RWC was determined by rapidly removing 10 leaves from each treatment, weighing them, and then immersing them in double distilled water to soak them for 24 h. Then the dry weight of the leaves was determined by oven-drying them to a consistent weight at 70 ^◦^C. The RWC was estimated using the following formula: RWC = (fresh weight − dry weight)/ (turgor weight − dry weight)^71^.

### Determination of fruit parameters

#### Determine quantity and quality

 The strawberries were picked to measure quantity and quality and brought to the lab in a refrigerated truck. The length and diameter of the fruits were measured using a digital caliper. Total marketable fruit yield (sum of early and late production) from January 1 to May 31 was recorded daily as grams per plant during the cropping cycle. A portable penetrometer was used to measure the firmness in a subset of three to four fruits from each treatment, and the results were reported in g cm^− 2^. An automatic digital refractometer was used to determine total soluble solids (TSS). In each treatment, the total acidity (TA) was determined by titrating 10 g of pulp and 10 mL of water with 0.1 mol L^− 1^ of sodium hydroxide until the pH reached 8.1 ^72^.

#### Antioxidant activity

 Zheng et al.^73^ provided the methodology for determining antioxidant activity. Ten milliliters of an ethanol solution containing 0.03 g L^− 1^ of DPPH was heated to room temperature and mixed with 0.1 milliliters of strawberry extract in ethanol. As a control, 0.1 mL of the extract was mixed with 10 mL of distilled water. Following a 30-minute reaction in darkness, the absorbance was recorded at 517 nm.

#### Anthocyanin content

 The Solovchenko et al.^74^ method was used with some modifications to the measurement of anthocyanin content. Anthocyanins were extracted using acidified methanol and chloroform. Extracts were obtained by adding 20 mL of chloroform-methanol (2: 1 v/v, acidified with 0.1% HCl) to the strawberry samples.

#### Ascorbic acid content

 Spectrophotometric methods were employed with potassium permanganate as the chromogenic reagent to ascertain the ascorbic acid level. To create the ascorbic standard calibration curve, 10 mL of each standard solution was added to a test tube along with 1 mL of KMnO_4_ solution (100 µg mL^− 1^), and the mixture was allowed to sit for five minutes. This standard solution’s absorbance was measured at 530 nm compared to a blank. Then, the graph of the absorption rate of solutions was drawn based on concentration.

#### Statistical analysis

 Data were analyzed using SAS Software (SAS Institute, 2004). Comparisons of means were performed using the LSD test at *P* ≤ 0.05.

## Electronic supplementary material

Below is the link to the electronic supplementary material.

Supplementary Material 1

Supplementary Material 2

## Data Availability

Data is provided within the supplementary files.
